# Photovoice for leveraging traditional, complementary, and integrative medicine amongst black adults to improve sleep health and overall health

**DOI:** 10.3389/fpubh.2024.1359096

**Published:** 2024-07-24

**Authors:** Rhoda Moise, Maurice Chery, Mykayla Wyrick, Ferdinand Zizi, Azizi Seixas, Girardin Jean-Louis

**Affiliations:** ^1^Department of Psychiatry, Center for Translational Sleep and Circadian Sciences, University of Miami Miller School of Medicine, Miami, FL, United States; ^2^Department of Public Health Sciences, University of Miami Miller School of Medicine, Miami, FL, United States; ^3^Media and Innovation Lab, Department of Informatics and Health Data Science, University of Miami Miller School of Medicine, Miami, FL, United States

**Keywords:** sleep, traditional, complementary, and integrative medicine, health equity, qualitative research, chronic disease, complementary and alternative medicine

## Abstract

**Introduction:**

Average adults are recommended to have 7–8 h of sleep. However insufficient sleep (IS defined as <7 h/nightly) is associated with increased risk of chronic diseases such as cardiovascular disease (CVD) and type 2 diabetes mellitus (T2DM). Traditional, complementary, and integrative medicine (TCIM), a burgeoning area of research and practice, leverages both modern and traditional approaches to improve health. Despite TCIM’s recognition as a tool to improve sleep and related outcomes, there is a gap in literature in addressing its impact among black individuals, who experience a disproportionate burden of IS and chronic disease. This qualitative study aimed to increase understanding of TCIM practices to overcome IS and overall health in black communities.

**Methods:**

Using photovoice methodology, a qualitative tool which applies community-engaged principles to produce culturally informed results through interviews and digital media, consented participants were recruited from Miami, Florida and (1) instructed to capture images over one week that communicated their TCIM to improve sleep and overall health on their mobile device; (2) interviewed using individual, semi-structured procedures to add “voice” to the “photos” they captured for ~20 min; and (3) invited to participate in follow-up focus groups for refined discussion and data triangulation for ~1.5 h. Both individual and focus group interviews were conducted over Zoom with recordings transcribed for formal content analysis using Nvivo software.

**Results:**

The sample included N = 25 diverse US black individuals (M = 37, SD = 13, range 21–57). Approximately a quarter of the sample were unemployed (*N* = 7) and majority were women (N = 21). Results highlighted five themes including: (1) natural wellness (sleep supplements, comfort beverages, aromatherapy, herbalism, outdoors); (2) self-care (self-maintenance, physical activity, spatial comfort); (3) leisure (pet support, play); (4) mental stimulation (mindfulness, reading); and (5) spiritual wellness (faith-based practices). Study results elucidate the heterogeneity of diverse US black individuals regarding sociocultural knowledge, beliefs, and behaviors.

**Conclusion:**

Addressing IS in black communities requires a comprehensive strategy that integrates cultural sensitivity, family and community dynamics, education, mental health support, and informed policymaking. Future studies should consider how sleep health literacy, stress appraisal, and coping strategies may vary by race/ethnicity for tailored intervention.

## 1 Introduction

Average adults are recommended to have 7–8 h of sleep (1–4). However insufficient sleep (IS) is associated with increased risk of chronic diseases such as cardiovascular disease (CVD) and type 2 diabetes mellitus (T2DM) and stems from various social and environmental determinants of health (e.g., mood and sleep disorders, shift work and extended work hours, unfavorable sleep environments) (5–7). Substantial evidence indicates significant racial/ethnic disparities in insomnia (IS), with Black individuals demonstrating a three-fold higher risk of IS compared to White counterparts (2, 8, 9). Notably, chronic diseases are the leading causes of death in the United States (US) and require a comprehensive treatment and management approach. However, there is an emerging body of literature seeking to address chronic conditions by investigating the utility of traditional, complementary, and integrative medicine (TCIM) (10–16). TCIM leverages a holistic approach to healthcare by combining conventional medical treatments with complementary and alternative therapies to treat the whole person with various modalities to promote wellness and address both physical and emotional aspects of health (17, 18). In fact, established literature recognizes TCIM practices, such as yoga and nutrition, as a tool to improve sleep health outcomes which in turn improve chronic health outcomes (10–17, 19).

Despite TCIM’s recognition as a tool to improve sleep and related outcomes, there is a gap in literature in addressing its impact among black individuals, who experience a disproportionate burden of IS and chronic disease (17, 19). Recent literature suggests typical health lifestyle behaviors, such as physical activity, do not confer the same health benefits among Black individuals as they do for White individuals (20–22). Underlying mechanisms contributing to these disparities include ecological and social determinants of health such as cultural differences in knowledge, attitudes, and behaviors; systemic inequalities in access to health resources; and population specific genetic variations (1, 2, 8, 19, 23, 24). Such differences may play a role in shaping the health outcomes associated with these behaviors across racial groups. Therefore, a more comprehensive exploration of these factors is essential to better understand and address the observed differences in health benefits derived from lifestyle behaviors among diverse populations (7, 25–31). TCIM practices for sleep and overall health optimization such as spirituality, herbalism, and meditation have been understudied in black individuals. Therefore, attempts to adapt TCIM interventions for black individuals without directly considering biopsychosocial pathways and experiences from black individuals may render efforts ineffective. Photovoice is a qualitative methodology which applies community-based participatory research (CBPR) principles in engaging members of the community to produce culturally informed results through interviews and digital media (32–34). Thus, a photovoice approach could be one method to facilitate communication between academic researchers and immigrant and minority communities to create the kind of partnerships that can address racial/ethnic disparities in sleep.

## 2 Materials and methods

### 2.1 Conceptual frameworks: the PEN-3 model and SHOWeD process

This study uses the SHOWeD photovoice protocol to engage black individuals with IS in semi-structured individual and group interviews grounded in cultural empowerment through allowing them to share their personal narratives with use of digital media (32–35). The PEN-3 Cultural Model provides a framework for data interpretation (32–35). Therefore, this study aims to (1) utilize photovoice to engage black individuals in the research process of understanding barriers and facilitators to incorporating TCIM in their lifestyle and (2) employs the PEN-3 Cultural Model to consider multilevel social and environmental determinants of health for public health planning and delivery. Overall, the anticipated results of the study could provide valuable insights into how TCIM can be effectively integrated into the healthcare landscape to better meet the needs of Black individuals, while also highlighting the importance of addressing cultural, social, and environmental determinants of health. To clearly provide participants study parameters, we initially focus on an asset-based model of current TCIM practices and then we will delve deeper into the scope of considering facilitators and empowerment by following theoretical approaches described below and detailed elsewhere (32–34).

The PEN-3 cultural model has been developed particularly for research in ethnic populations for cultural sensitivity and specifies three dimensions including the following: (1) cultural identity (person, extended family, and neighborhood); (2) relationships and expectations (perceptions, enablers, nurtures); and (3) cultural empowerment (positive, existential, negative) (32, 35, 36). These dimensions aid in organizing and interpreting data. As theorized in the PEN-3 Cultural Model (35) as well as other ecologically oriented models, multiple levels of influence shape one’s health. For instance, the cultural identity aspect of the PEN-3 cultural model assesses the individual at a personal level, communal extended family level, and societal neighborhood level. The dimension of relationships and expectations includes one’s perceptions, enablers, and nurturers and provides theoretical underpinnings for analysis of various sociodemographic variables and both individual (perceptions) and interpersonal (enablers and nurturers) factors (35). Positive, existential, and negative elements that shape one’s cultural empowerment.

Wang and Burris’s “SHOWeD” process for photovoice data (Table 1) encompasses a series of questions to guide conversation including (1) What do you See here? (2) What is really Happening here? (3) How does this relate to Our lives? (4) Why does this situation, concern, or strength Exist? (5) What can we Do about it? Through modifying the power dynamic of dominant researcher examining vulnerable participant to be more collaborative, this study allows participants to exercise autonomy in artistic expression of scientific measure through photovoice (33, 35). SHOWeD provides a scientific guideline to data acquisition and interpretation whereas the PEN-3 Cultural Model serves as a thinking tool to aid interpretation and implications of health behaviors in ethnic populations.

**Table 1:** Photovoice nine-step study procedures.

Group (N)	Recruit/Consent/Cameras (1, 2, 3, 4, 5, 6)	Pictures (7)	Discussion (8)	Dissemination (9)
G1 (8)	July 19–21	7 days	July 28	December 15
G2 (8)	July 21–24	7 days	July 31	December 15
G3 (9)	July 24–28	7 days	August 2	December 15
FG1 (6)	–	–	August 4	December 15
FG2 (6)	–	–	August 4	December 15

All dates are 2023; G = group and FG = focus group; Wang and Burris recommend steps are as follows: (1) select and recruit target audience; (2) recruit a group of photovoice participants; (3) introduce the photovoice methodology to participants; (4) obtain informed consent; (5) pose an initial theme for taking pictures; (6) distribute cameras to participants and review how to use them; (7) provide time for participants to take pictures; (8) meet to discuss photographs; (9) plan with participants a format to share photographs and stories with leaders.

### 2.2 Study recruitment and data analysis

In recruiting participants for the photovoice study, a purposeful sampling strategy was employed to select individuals from the ongoing R01 study, Determinants of insufficient sleep among black individuals and effects on disparities in health outcomes (ESSENTIALS; R01HL142066–06). This strategy was chosen to ensure that participants had relevant experiences and insights related to sleep health within the target population. To address potential biases, efforts were made to diversify the sample by considering factors such as age, gender, socioeconomic status, and geographic location. The sample was deliberately constructed to encompass the diverse Black population residing in South Florida, thereby ensuring representativeness within the study cohort. Efforts were made to encompass both US-born and foreign-born individuals, reflecting the demographic composition of South Florida. Additionally, steps were taken to establish the generalizability of the findings by conducting in-depth analyses of the data, triangulating findings with existing literature, and engaging in ongoing dialogue with community stakeholders to validate and contextualize the results. These measures were aimed at enhancing the validity and reliability of the study findings, thereby strengthening the credibility of the research outcomes.

Throughout the photovoice study, we implemented CBPR principles by actively involving community members in decision-making processes, ensuring their voices and perspectives were central to the research (32, 37). For participant recruitment, we collaborated closely with community organizations and leaders to identify and engage individuals who best represented the community’s diversity and needs (34, 38). Moreover, in terms of study design, community members were consulted at various stages to ensure the research questions, methodologies, and outcomes were culturally relevant and aligned with community priorities (33, 39).

The study team offered comprehensive training sessions for participants on how to effectively utilize photovoice as a research tool similar to previous photovoice study standards (32, 34, 38). These sessions covered topics such as the purpose of photovoice, ethical considerations, technical aspects of photography, and guidelines for selecting and captioning photographs. Participants were given ample opportunities to practice their photography skills and engage in discussions to ensure they understood the objectives of the project and how their contributions would be utilized (33, 34). Additionally, ongoing support and guidance were provided throughout the study to address any questions or concerns that arose. Participants were given a week to capture five photographs that communicated their natural, integrative practices to improve sleep and overall health. Participants were then asked to participate in a follow-up individual, semi-structured interview to add “voice” to the “photos” they captured for approximately 20 min. Individual interviews created a safe space for participants to share their insights for the first time within the study (32). Information redundancy or saturation was ascertained when no additional barriers or themes were articulated by participants (40–43).

Study personnel invited participants who were interested and amenable to secondary focus group interview dialogue to further discuss and triangulate data, a technique to assure validity through usage of more than one data collection method. Two focus groups spanned 1–1.5 h with six participants each. Targeted themes of focus groups included knowledge, beliefs, and attitudes about TCIM for sleep (e.g., spirituality, herbalism, and meditation), as well as mediating and reinforcing factors associated with healthful sleep behavior (e.g., families, workplaces, religious affiliations, or community and societal structures). Additional validation techniques included a member checking method where focus group participants clarified the initial individual interview results as well as keeping record with detailed audit notes in addition to audiovisual recording (40, 41, 44). Result reliability was further established through research techniques including prolonged field engagement by means of recruitment through an existing federally funded large research study to develop familiarity and trust with the participants (40, 41). Both individual and focus group interviews were conducted over Zoom with recordings transcribed for formal content analysis using Nvivo software.

Participants identified and described images that best captured their natural practices for improving sleep and overall heath. For comprehensive inquiry, participants also were given the opportunity to articulate any practices which were difficult to photograph. Initial individual interviews offered understanding of significant photographs which served as the codes for thorough content analysis and focus group interviews provided further discussions and data validity. Codes were organized into major themes by two research personnel independent from the corresponding author who conducted the interviews to avoid biased content analysis using NVivo qualitative data analysis software (33, 34, 41, 45). In order to optimize the interpretation for ecological implications, investigators employed a combination of manual inductive methods involving document review and organization by study personnel and deductive techniques, which included machine assessment using software like NVivo to analyze word frequencies in transcripts (41, 43). The final selection of images was guided by the principles of PEN-3, specifically focusing on cultural identity, relationships, expectations, and cultural empowerment, as guided by relevant literature (32, 35, 36).

## 3 Results

### 3.1 Participant characteristics

The sample (Table 2) included participants who all self-identified race African American or Black (N = 25) with history of insufficient sleep (IS) but not diagnosed with a sleep disorder. Our sample included diverse US black individuals (N = 8 foreign born) who were on average 37 years of age (SD = 13, range 21–57). Further, over half the participants had at least one foreign born parent (N = 16). Additionally, several participants spoke a language other than English at home (N = 10). Approximately a quarter of the sample were unemployed (N *=* 7). Of those employed (N = 18), participants reported occupations including the health sector, administration, homemaking, and education. The majority of participants also reported personal, or family history of chronic conditions related to sleep (N = 18). These chronic conditions spanned cognitive, cardiometabolic, inflammation issues (e.g., dementia, epilepsy, stroke, diabetes, cancer, hypertension, high cholesterol, asthma, arthritis, edema, substance addiction).

**Table 2:** Characteristics of photovoice participants *N* = 25.

N	Age	Sex	Ethnicity*	Foreign born parent	Language spoken at home	Employment status	Familial chronic condition
1	49	F	Black	Y	E	Unemployed	Y
2	29	F	AA/Ethiopian	Y	E	Administrator	Y
3	55	F	Black	N	E	Unemployed	Y
4	57	F	Black	N	E	Neighborhood worker	Y
5	22	F	Nigerian-A	Y	E and Igbo	Medical scribe	Y
6	29	F	AA	N	E	Community health worker	Y
7	37	M	AA	N	E	Unemployed	N
8	32	M	Afro-CB-European*	Y	E, Patois, Gypsy	Community assoc. manager	Y
9	51	F	CB	Y	E	Auditor	N
10	23	F	Black	N	E	Unemployed	N
11	41	F	Black	N	E	Health project coordinator	Y
12	24	F	Liberian	Y	E, Colokwa, Bassa	Student and researcher	Y
13	21	F	Rwandan*	Y	E, F, Kinyrwanda	Student	N
14	25	F	Nigerian	Y	E, Igbo	Student	Y
15	53	F	American	N	E	Housewife	N
16	24	M	Nigerian-A	Y	E, Ibibio	Unemployed	Y
17	51	F	St. Lucian*	Y	E	Teacher	Y
18	55	M	AA	N	E	Unemployed	Y
19	27	F	Haitian*	Y	E, S, Creole	Medical student	Y
20	21	F	Jamaican*	Y	E, Jamaican Creole	Student	N
21	25	F	Haitian*	Y	E, F, S, Haitian Creole	Researcher	Y
22	30	F	Black	Y	E, S	Unemployed	N
23	35	F	Black*	Y	E	Grants analyst	Y
24	56	F	Haitian*	Y	E, F, S, Creole	Social worker	Y
25	27	F	Black	N	E	Researcher	Y

Sex: F, Female; M, Male; Ethnicity: *, foreign-born; AA, African American; B, Black; -A, hyphenated American; CB, Caribbean; Language Spoken at Home: E, English; F, French; S, Spanish; Familial Chronic Conditions: Y, Yes; N, No.

### 3.2 Qualitative content analysis: individual interviews

This study’s research produced five themes (Table 3) with parallel subthemes: (1) natural wellness (sleep supplements, comfort beverages, aromatherapy, herbalism, outdoors); (2) self-care (self-maintenance, physical activity, spatial comfort); (3) leisure (pet support, play); (4) mental stimulation (mindfulness, reading); (5) spiritual wellness (faith-based practices). The development from the individual interviews guided the dialogue for the focus groups.

**Table 3:** Themes, definitions, and subthemes of photovoice for leveraging TCIM amongst black adults to improve sleep health and overall health.

Theme	Definition	Subthemes
1. Natural wellness	Engaging with natural and herbal elements and practices to promote holistic health and well-being.	Sleep supplements, comfort beverages, aromatherapy, herbalism, outdoors
2. Self-care	Activities and practices that focus on nurturing one’s physical and mental well-being.	Self-maintenance, physical activity, spatial comfort
3. Leisure	Activities and elements that provide relaxation, enjoyment, and recreation.	Pet support, play
4. Mental stimulation	Engagements that challenge, stimulate, or soothe the mind, enhancing cognitive and emotional health.	Mindfulness, reading
5. Spiritual wellness	Practices and experiences that nurture the spirit, fostering a sense of peace, purpose, and connection.	Faith-based practices

### 3.3 Qualitative content analysis: focus group interviews

The analysis identified five key themes with associated codes or subthemes, outlining strategies for personal well-being: (1) Natural Wellness: This includes the use of sleep aids like melatonin, comfort drinks (e.g., milk, hot chocolate), herbal remedies (cannabis, tea), aromatherapy (candles, diffusers), and spending time in nature. (2) Self-Care: This theme covers personal grooming and relaxation techniques (skin care, massage, cooking, napping, solitude), physical exercises (cardio, yoga, biking), and creating a comfortable space (through soothing audio and sleep-enhancing accessories like cooling pillows). (3) Leisure: Activities here involve interaction with pets (cats, dogs, birds), and engaging in play (video games, musical instruments, music). (4) Mental Stimulation: This includes practices for mental clarity and engagement such as mindfulness (breathwork, journaling), and reading for leisure and knowledge expansion. (5) Spiritual Wellness: The theme is centered around engaging in faith-based practices as a means to spiritual grounding and peace. Results from individual interviews further guided discussion in focus group interviews.

### 3.4 Natural wellness

**Figure 1:**
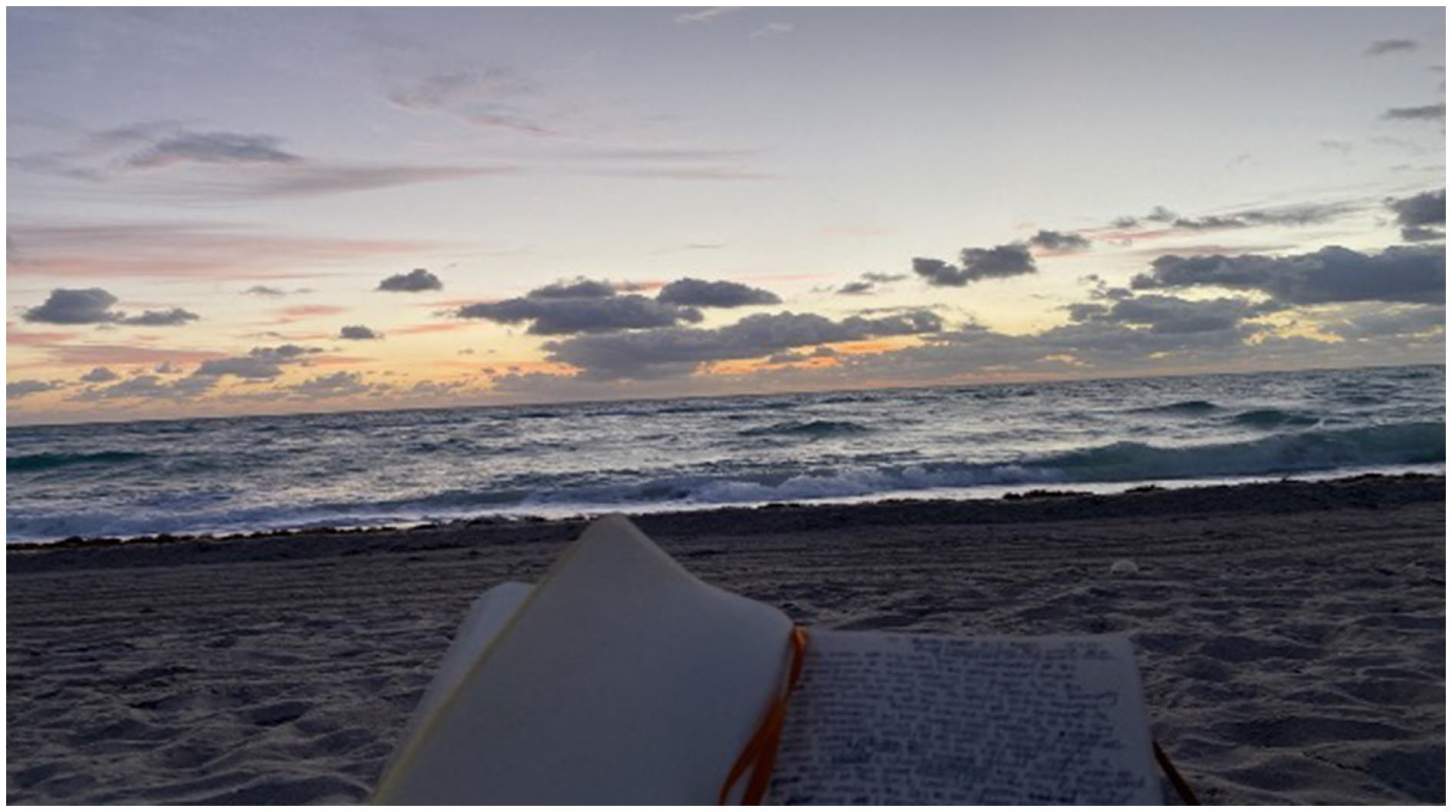
“I truly believe that this is a necessity for wellness, and it’s much needed for just the human way of life to connect with the earth, connect with the sun, the ocean, plants, we are all one this earth together”.

**Figure 2:**
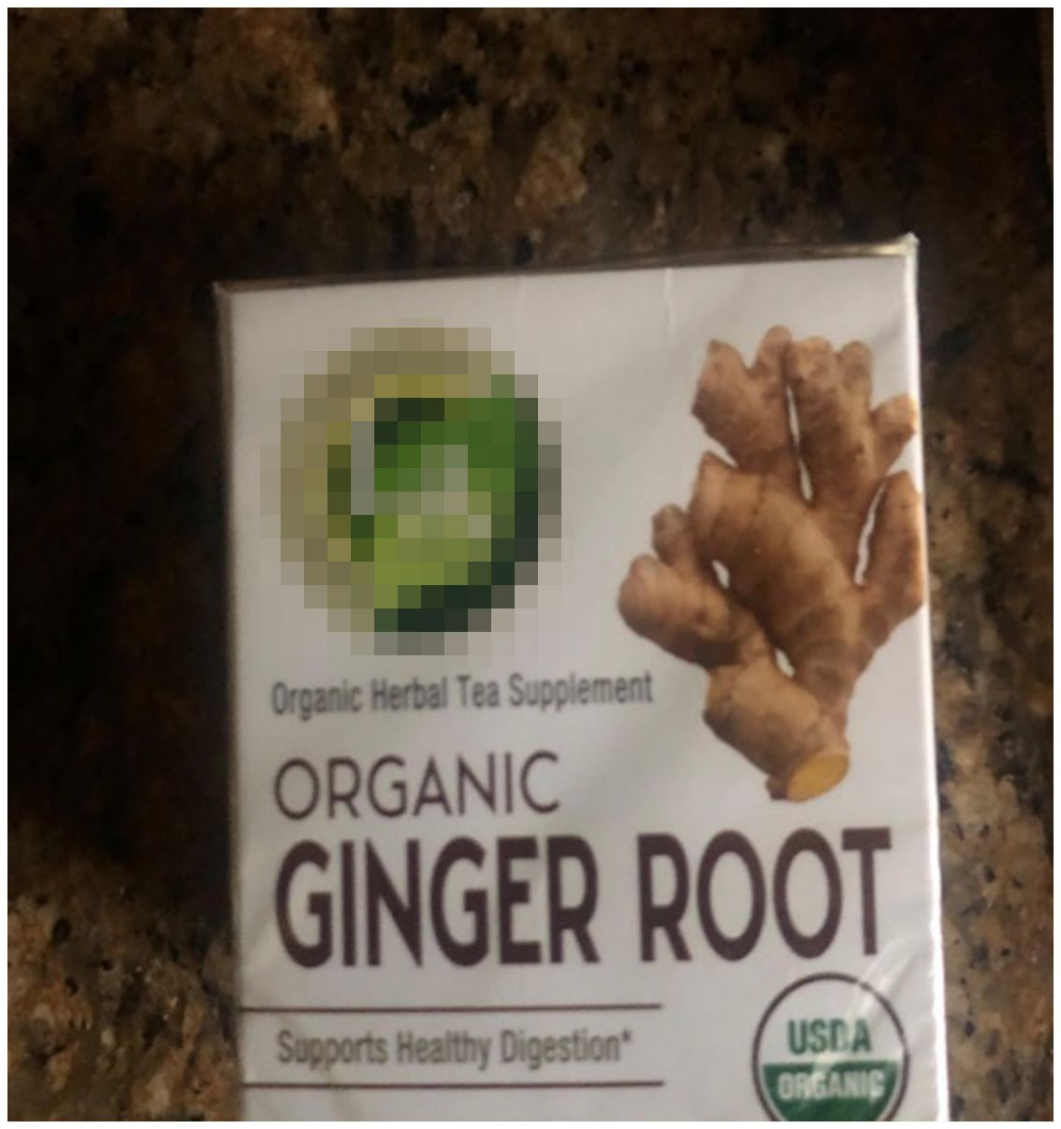
“[I] started to make some infused tea with [herbs] as a night tea before sleeping, after I eat. Because I’ve realized it not only helps with sleeping afterwards but also it kind of improves my digestion”.

Participants underscored the importance of natural wellness in their overall health and sleep practices (Figure 1). The analysis revealed a clear preference for natural sleep aids among the participants. These were not merely chosen for their effectiveness but also for their connection to nature’s inherent restorative qualities. The habitual intake of warm beverages before bedtime was highlighted as a simple yet meaningful routine that participants believed prepared their mind and body for rest. Herbal remedies were valued not just for their direct impact on sleep but also for their broader contribution to well-being, suggesting a holistic approach to health (Figure 2). Aromatherapy was consistently mentioned as a method for creating a sleep-conducive environment, with natural scents being used to promote relaxation and tranquility. Furthermore, exposure to the outdoors was frequently associated with improved sleep, pointing to the therapeutic potential of the natural environment. For example, a participant shared:

*“I would say it’s supplements that different people use depending on sleeping difficulties that they might face because some people often might find it hard to get sleep at night, or they might find it hard to stay asleep. So, I know that chamomile tea, when I put two-three baggies in there, it kinda – it knocks me out. It puts me to sleep. It just – I don’t know. I just close my eyes, and I’m gone. It works.”*

### 3.5 Self-care

**Figure 3:**
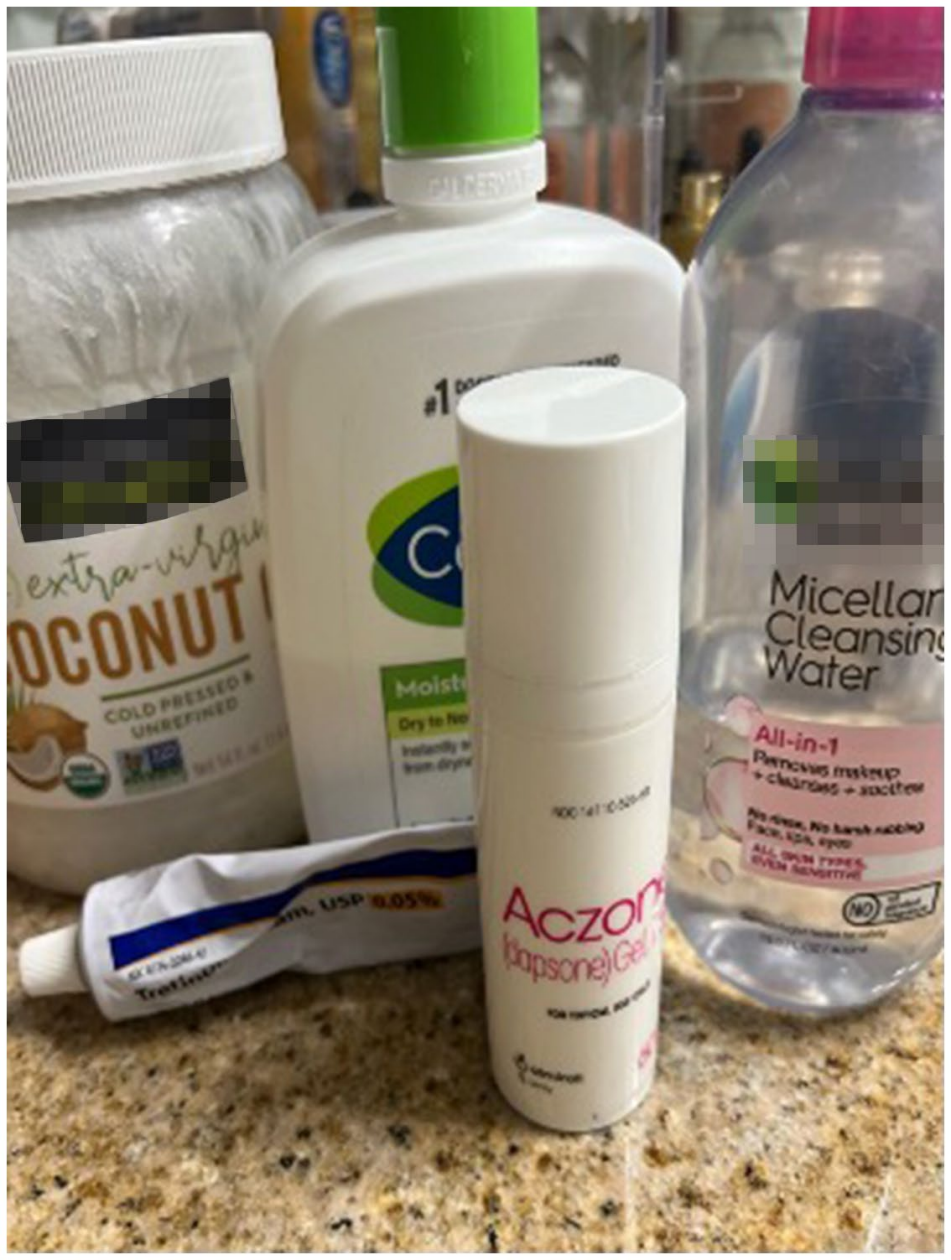
“After I shower, of course, I use my facewash in the shower, and then I use the micellar water just to clean up, make sure my skin’s extra clean… And I think just doing that-because I do not really do that in the morning, but doing that at night just helps wind me down and lets me know that it’s time to get ready for bed”.

**Figure 4:**
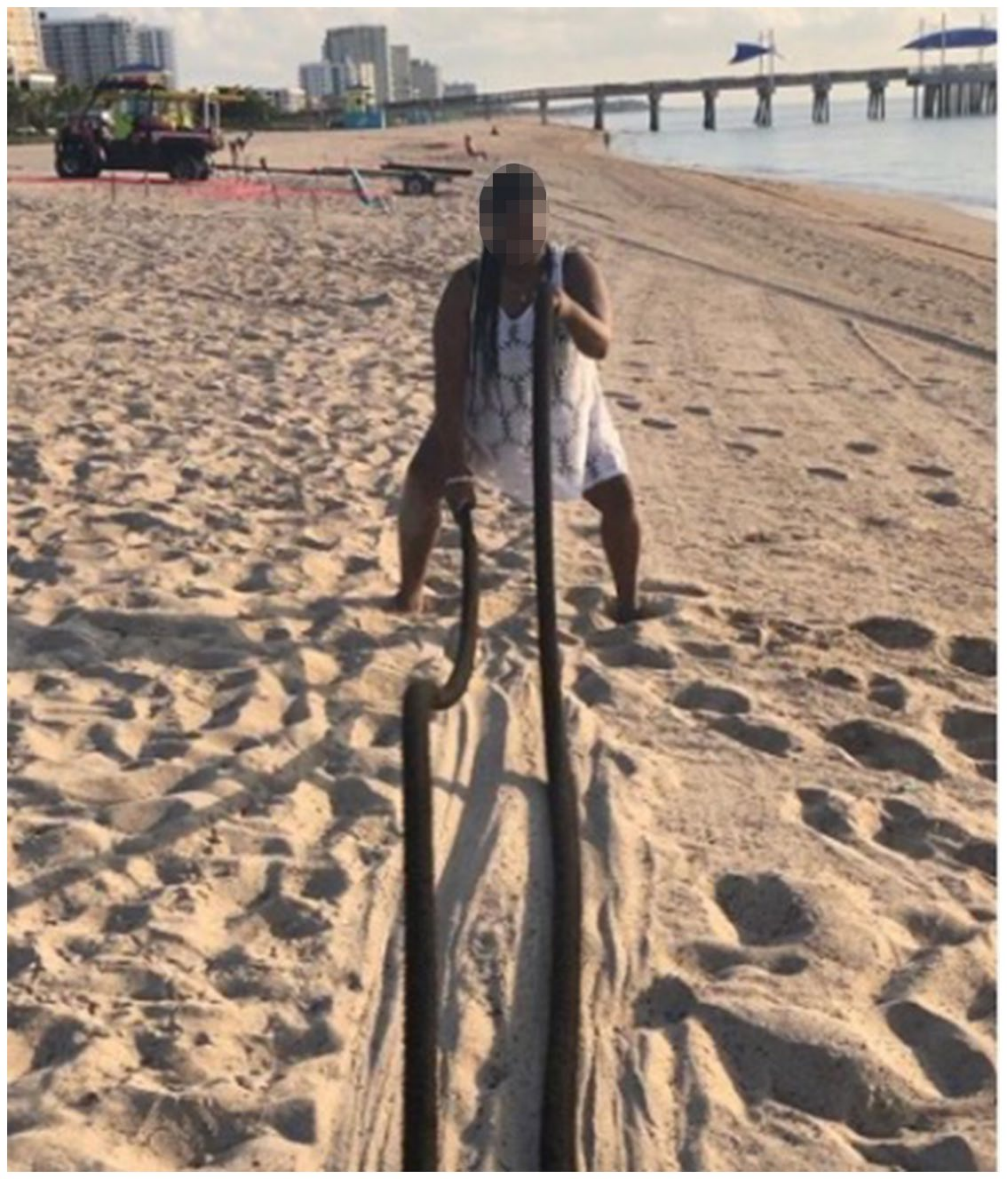
But I do know that [exercise] right there is one of the main things that helps me also get tired throughout the day. Because after I’ve burned over 3,000 calories, I’m super exhausted. So [I’m] able to just lay down and try to relax and go to bed”.

Participants considered self-maintenance activities to be of paramount importance, extending well beyond mere routine. Participants saw these practices as vital in establishing a pre-sleep ritual that transitioned them from wakefulness to sleep (Figure 3). Physical exercise, for example, was highlighted as a means to dissipate stress and physical discomfort, which participants felt were barriers to restful sleep (Figure 4). The individual customization of their sleep environments further underscores the participants’ proactive approach to self-care. They did not merely seek comfort but engaged in a thoughtful selection of elements such as auditory stimuli, which might include soothing sounds or white noise. Participants chose these elements to address individual sensory preferences and sensitivities, which were recognized as significant contributors to sleep disruption or facilitation.

Moreover, participants often spoke of self-care in terms that suggested a holistic engagement with their well-being. It was not just a single action but a suite of behaviors that cumulatively created a conducive atmosphere for sleep.

*“Well, I feel it [yoga] keeps my irritability or the – how erratic my thoughts are, like the level of frenzy in my thoughts, and so, when I’m just calmer in general, it’s easier to just lie down. Anything that creates me more, gives me more peace, because my day is so running gun and very anxiety inducing. Anything allows me to kind of be still and kind of just detach from my outward responsibilities brings me more peace and more peace calms me down and ensures better sleep.”*

### 3.6 Leisure

**Figure 5:**
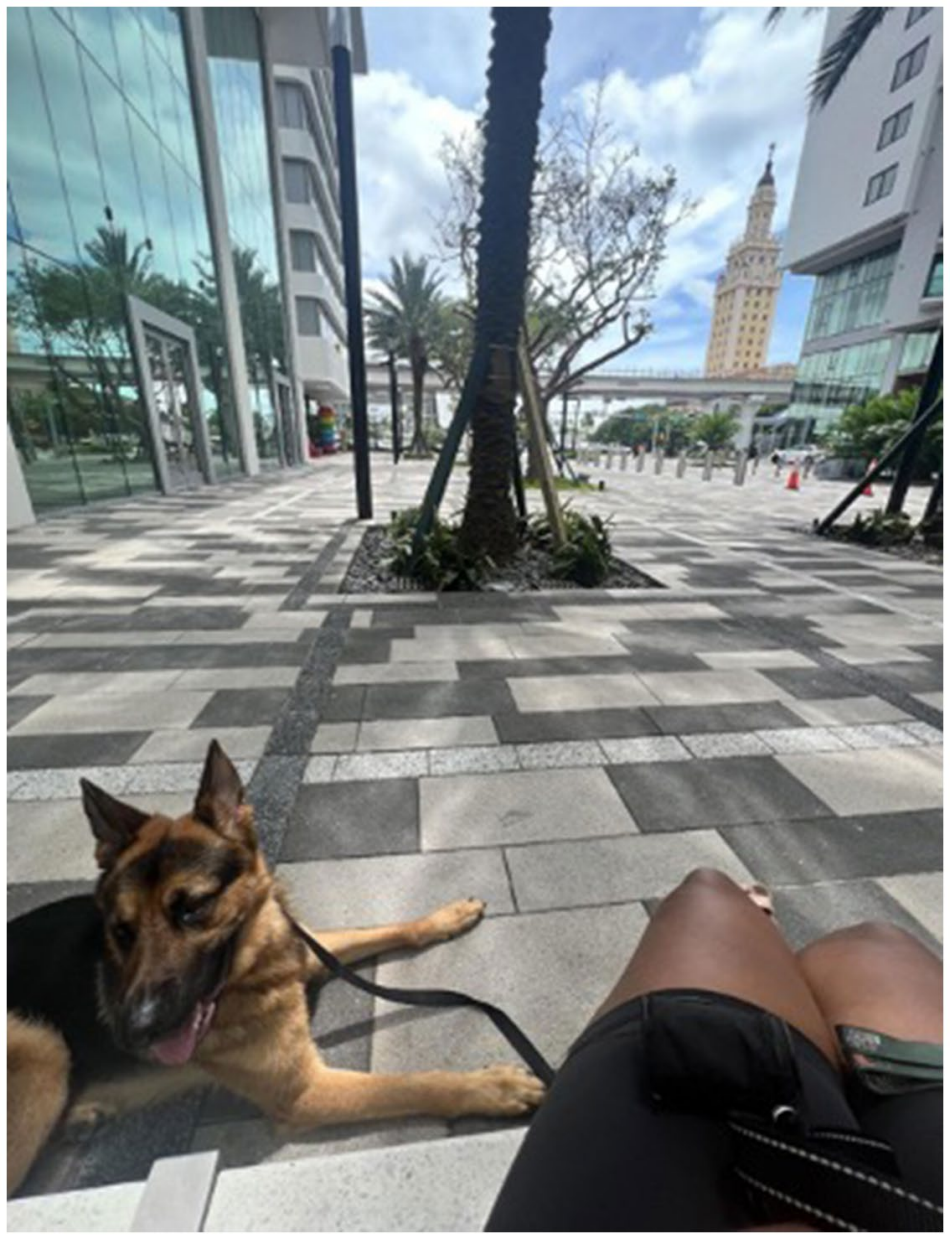
“I do know that dogs they love to cuddle…And it helps you fall asleep. And it also makes you feel safer, too. So you can have a peace of mind that you have a best friend next to you and nothing’s gonna happen to you”.

**Figure 6:**
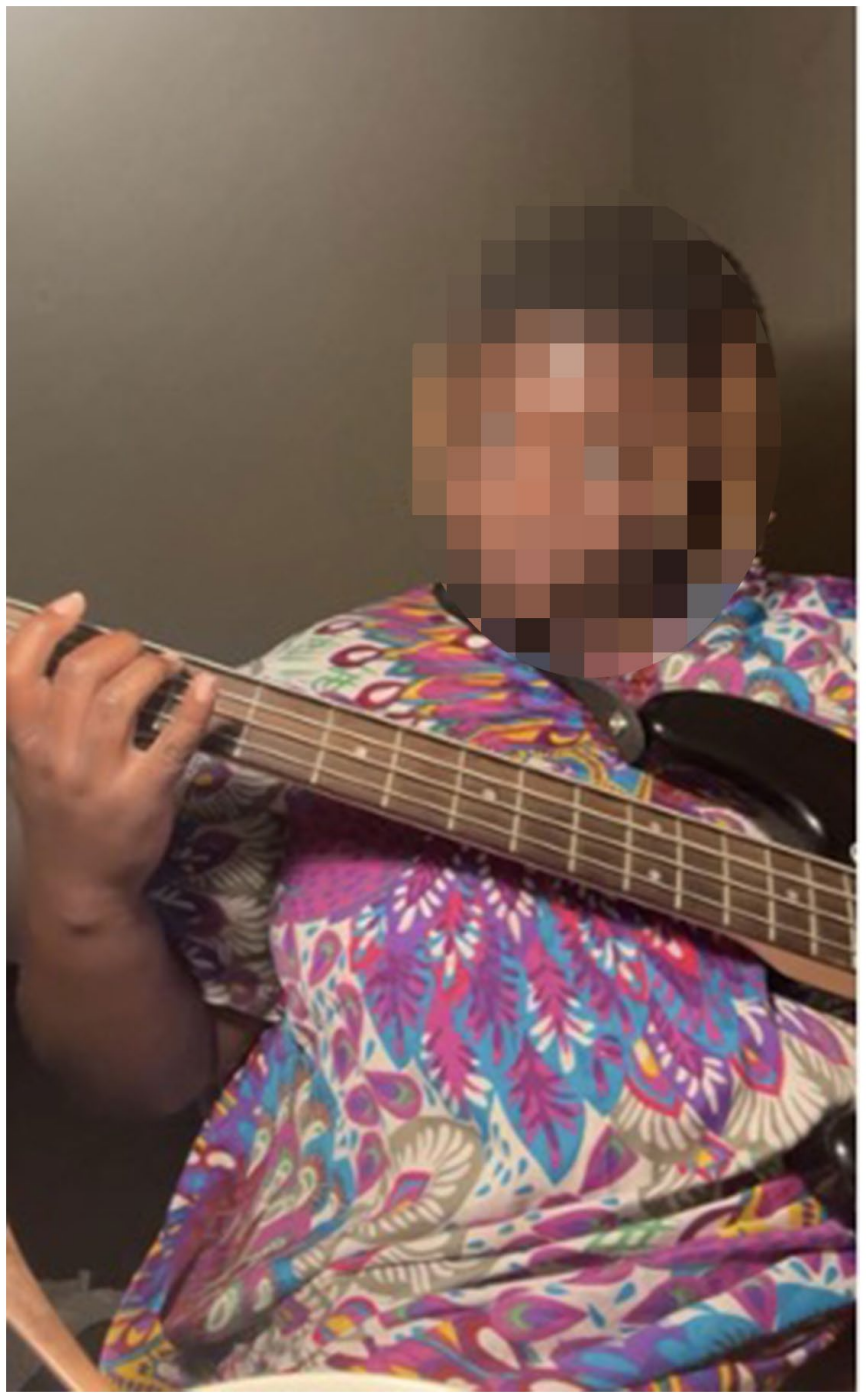
“I was very concentrated, and very motivated playing this instrument, but just getting into a routine, practicing to kind of wear myself out so I can fall back asleep again, and maybe doing this for maybe for I wanna say 30 to 45 min got me sleepy enough to revisit the bed”.

Participants detailed how leisure activities were crucial in unwinding their minds, serving as a prelude to their nightly sleep. They spoke of the comfort and emotional support derived from the presence of pets, emphasizing how they provided a unique form of companionship that allayed their anxieties and facilitated a sense of protection as they drifted off to sleep (Figure 5). Furthermore, they shared how engagement in recreational activities (playing games, strumming a guitar, listen to music) acted as effective transitional activities (Figure 6). These pursuits allowed them to detach from the pressures and stress of their daily routines, serving as a deliberate shift from wakefulness to a state primed for rest. Participants also elaborated on how these leisure activities, by offering a joyful and satisfying end to their day, played a significant role in shaping their mood and readiness for sleep. They described this time as essential in signaling to their bodies and minds that the business of the day had ended, making way for the calm of the night, setting the stage for a restorative sleep.

*“But I don’t know if you have pets, but I feel like it’s totally true just snuggling with an animal, cats, they purr. I feel like that relaxes me…. So, I really think it’s just a comfort thing to have someone that’ll listen to you and not judge you, and just love you regardless of what you’re going through.”*

### 3.7 Mental stimulation

**Figure 7:**
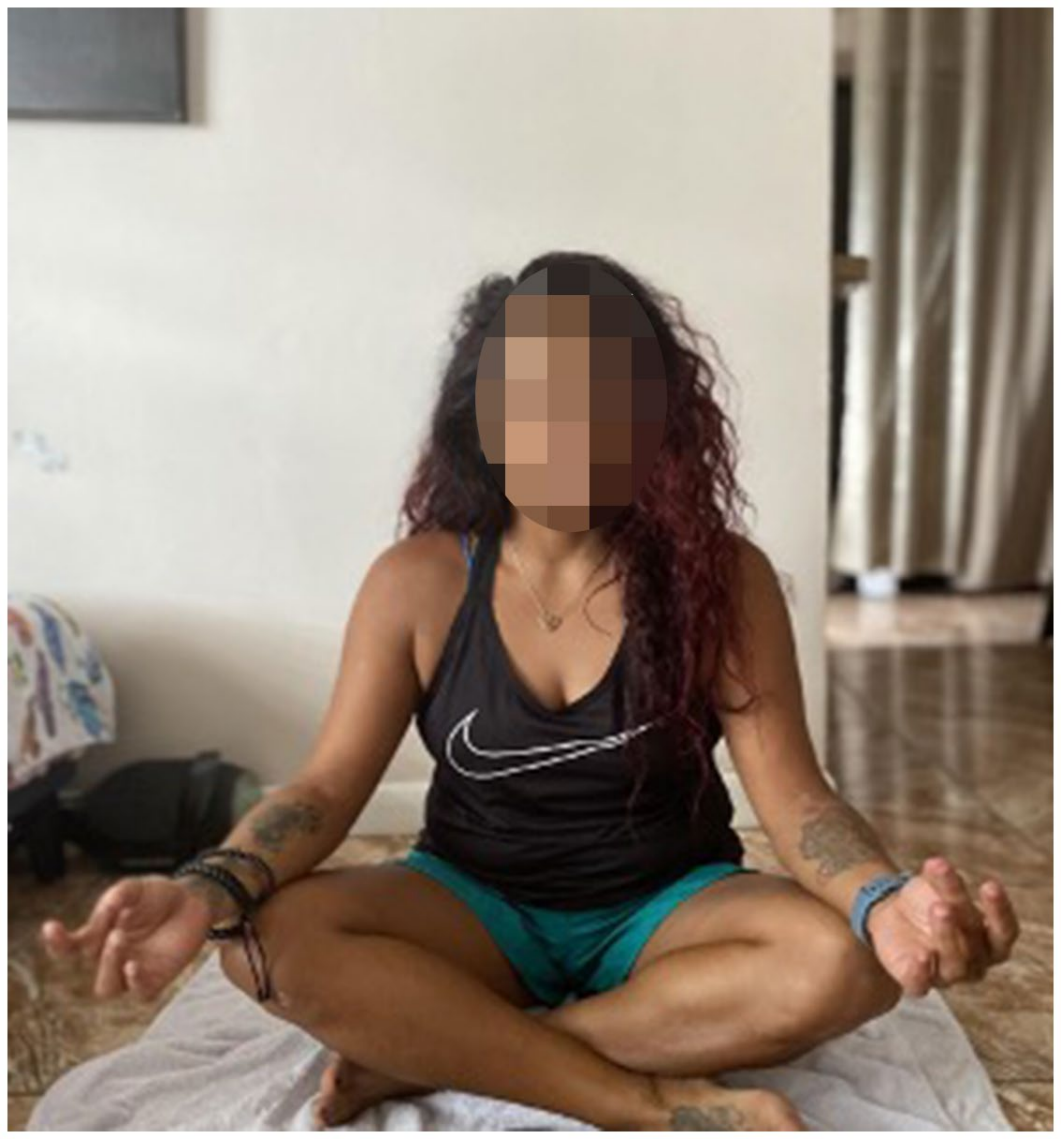
“I think people are kind of more accepting of this in everyday life. We do this and we do not realize it, whether it be breath work, or I’m stressed. We’re just like let me take a deep breath. Let me take 10 s for myself. This is something that we are not as conscious of but we are doing and making it more conscious…”.

**Figure 8:**
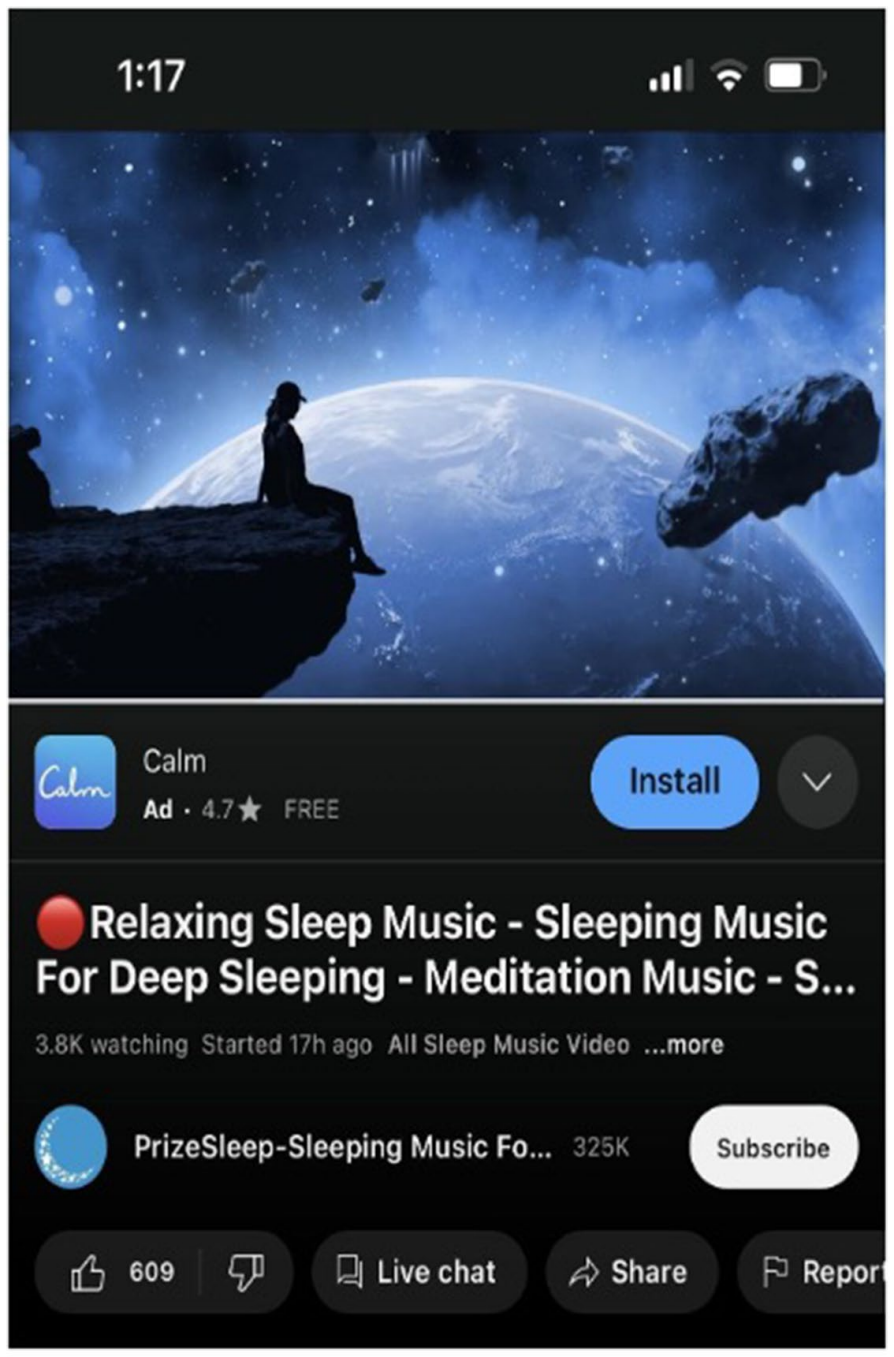
“I think it really just depends on how I’m feeling. Most of the time I do use the brown noise just because I feel like it’s a little bit more subtle. But if I do wanna drown out more noise, I feel that the white noise is a little bit louder, but it’s still comforting enough for me to fall asleep to …”.

The participants recounted that their deliberate engagement in mental stimulation activities was a strategic measure to prepare their minds for rest. They described their mindfulness practices, which included measured breathing and focused attention, as essential evening rituals to dispel the day’s mental clutter and foster a state of serenity conducive to sleep (Figure 7). The participants also emphasized how the act of reading served as a mental gateway, offering them an escape into narratives that distanced them from their own pressing thoughts, effectively quieting their minds. This narrative journey through books was articulated as a purposeful pre-sleep routine, which allowed them to transition from the complexity of reality to the simplicity of sleep. Through these practices, the participants illustrated a thoughtful process of mental deceleration, one that supported their overall sleep hygiene by gradually diminishing cognitive arousal and leading them gently towards sleep (Figure 8).

*“I think breathing is really important that we don’t focus enough on. It’s good for our body and just how our body works physiologically, but also just contributing to mindfulness and aiding for peaceful periods. I think meditating is very necessary because we need to slow down from our very fast-paced lives….”*

### 3.8 Spiritual wellness

**Figure 9:**
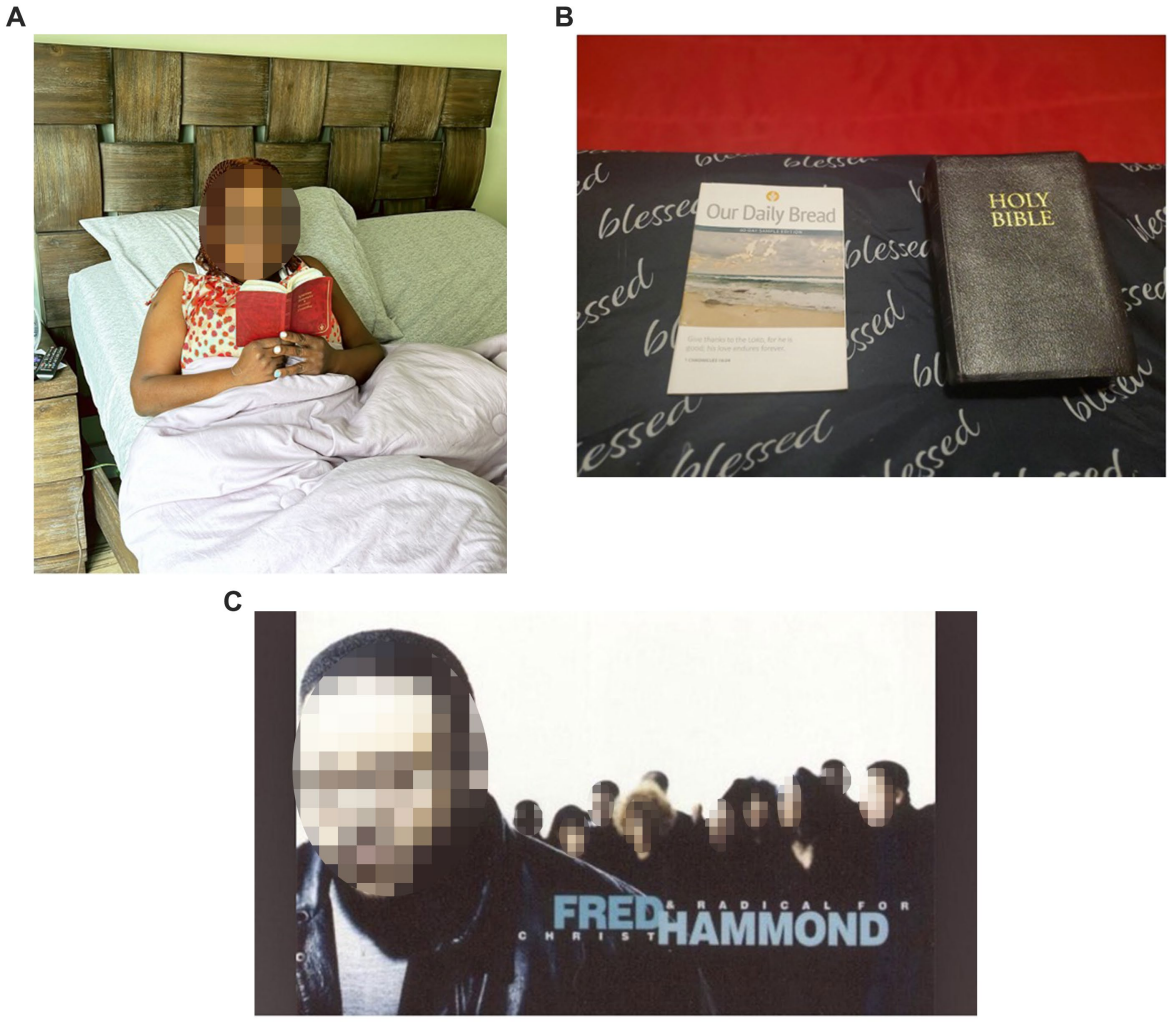
**(A–C)** “[When] things get rough, there’s always a higher power that we need to reach out to help us out with the things that we cannot solve by ourselves. I think we are all, innately, people always want something bigger than themselves to give them meaning, to give them comfort, to guide them in their everyday life, no matter what religion, spirituality, whatever practices you do, we are all wanting that same thing…”.

The participants conveyed that their spiritual practices were deeply intertwined with their sleep experience, attributing a significant role to these practices in achieving a restful night. They articulated that their faith-based activities were not mere habits but integral components of their nighttime routine, providing them with profound calm and a purposeful conclusion to their day. These spiritual engagements, as described by the participants, acted as a prelude to sleep, fostering an atmosphere of peace that was vital for transitioning into a state of rest (Figure 9). The participants regarded these practices as a grounding force, one that not only readied them for sleep but also enriched their lives with a deeper sense of fulfillment and tranquility.

*“I believe because we just need guidance. We don’t know everything. And these practices have been going on for so many years going back… So, prayer for me is, yeah, the actual praying to God, but also just praise. So me being in the church, being in the Word, so just reading and listening to the pastor. But I think throughout the day is more little conversations with God about just my everyday life or things that I’m going through and feeling.”*

### 3.9 Cultural influence

Notably, the sample included an array of cultural heritages of diverse US black individuals both including U.S.-born and foreign-born black individuals. Further, over half of the sample reported having at least one foreign-born parent. Participants geographically reported linages from both West and East Africa, throughout the English- and French-speaking Caribbean, and various regions of the U.S. Participants were specifically prompted to share their perspectives on how their culture may influence sleep health and related behaviors. Although some stated they found no relevance, nearly a quarter of the sample felt their culture significantly impacted (*N* = 8). Namely, Caribbean participants referenced cultural practices using herbalism or plant medicine for sleep.

*“It’s a requirement. You have to have tea before bed… So, I think that’s something very specific to … Caribbean culture… So, that is definitively like an TCIM practice… A lot of the herbal and naturopathic routes that I kind of default to when trying to find relief in sleep come from being of Haitian heritage. If I tell my mom that I can’t sleep, before she thinks about melatonin, she’ll tell me… [about] that specific tea with those leaves that are supposed to help with sleep.”*

Insight into African American familial heritage intersected with religion and social determinants of health also were articulated:

*“I cannot really get anyone to go work out with me. And that’s when I realized culturally – you know, my friends are sitting there talking about our metformins, and all of us are on it. All of us gotta take Ozempic … We’re all tired… most African Americans are brought up with soul food. It’s really heavy sometimes, and sometimes it makes you restless, you know, the way that you eat … And I was just never brought up to know about exercise at all … I never seen my family members exercise… I saw my family go to church all the time, and we eat at church, and we get really tired and don’t exercise the food off, and all of that weighs on your weight. That’s how I became diabetic. And it weighs on your sleep apnea, your breathing. All of that I think plays a part in the way that we sleep …”*

Furthermore, participants with African, Caribbean, and African-American heritage all noted cultural perspectives as influential. Key elements included cultural norms, familial responsibilities, and gender roles. For instance, when referencing immigrant heritage and sleep health literacy, insights included:

*“I know where my parents are from, they sleep for like, probably, six hours, and then, they’re up for the whole day. That’s just like everybody in the house does that when I’m back in Nigeria… my family is hardworking, and so they’re not too keen on me sleeping the day away. Not that I’m against that. I mean, I could say I’m 100% in agreeance with it.”*

Additionally, when discussing familial responsibilities from a cultural perspective, participants shared:

*“So, culture, depending on where you’re from, I mean, there might be certain expectations put on certain roles, so father, mother, or husband, or wife, or child dictating how much you should sleep, determining or dictating how long you should be awake, how much you need to do a day, and so that’ll effect how much you’re sleeping, how much you will be able to sleep in”*

In regard to gender roles, participants shed light on similarities in African-American culture stating:

*“I think it’s the same for African American women as well as the Caribbean culture. We’re just supposed to be strong. It takes a toll on you… And I think the stress of not letting go and trying to multi-task and control everything that goes on in the house, at school, at work, takes its toll on me as a person… it’s that type of thing that kind of makes you just have [anxiety and tiredness].”*

When discussing cultural perspectives as an immigrant, participants described the benefit of therapy for mental health:

*“Therapy has helped me not let it have such an effect on me. Because at least with my family, and I know with a lot of other Caribbean families as well, especially that I’m the oldest, there was a lot of pressure put onto me. The first to go to college. The first to move out. The first to buy a place on their own. I was the first even grandchild or cousin to do a lot of this, so I had a lot of pressure on me to keep doing better. So, I think going through therapy and knowing that it’s not always a competition, that everyone goes at their own pace, it’s really helped relieve a lot of that stress. And I think that has helped my sleep tremendously.”*

### 3.10 Challenges in sleep health equity

**Figure 10:**
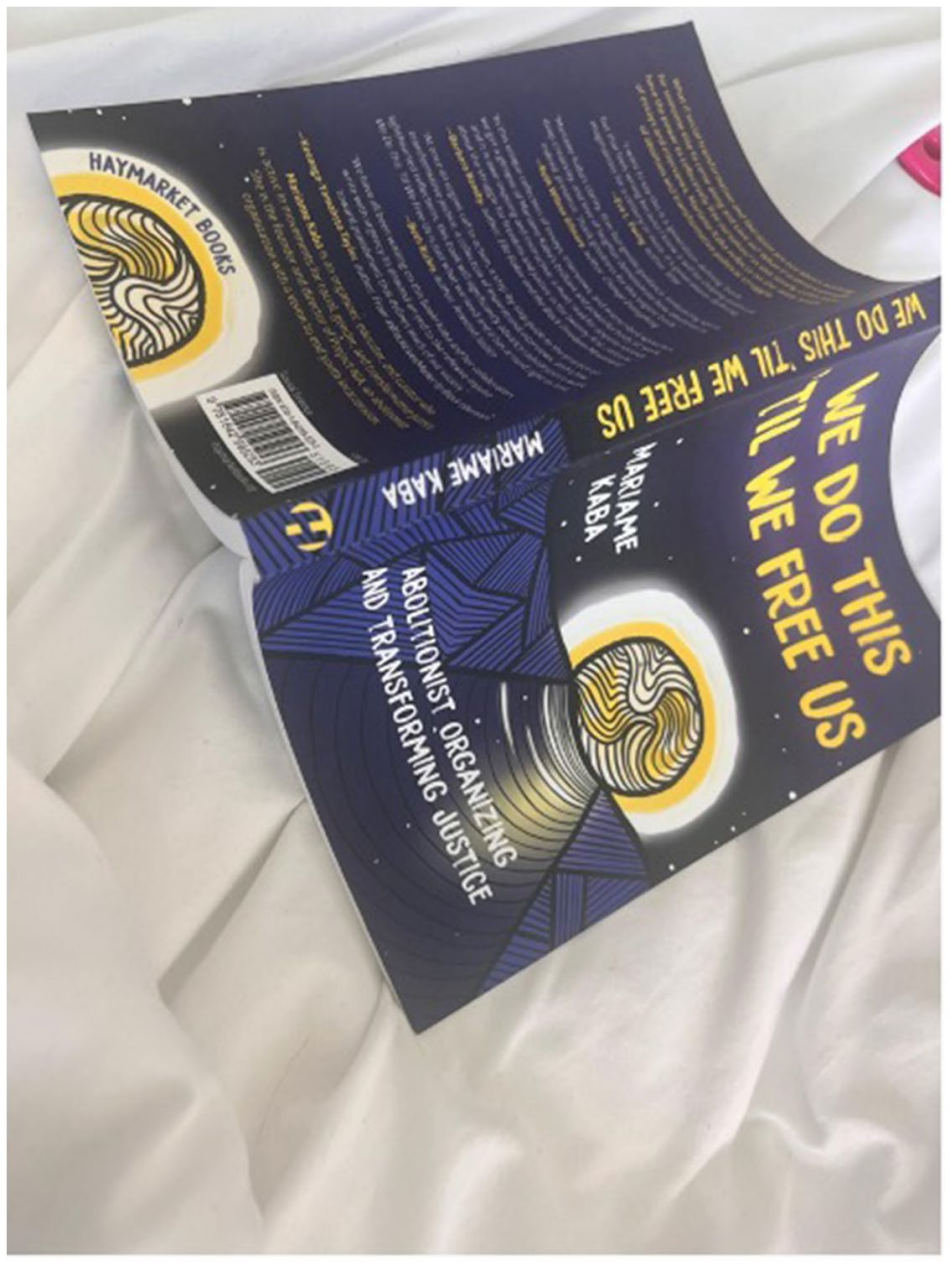
“I mean, racism, discrimination pretty much [impact black people’s sleep], because you see it in every area. Whether it’s like you are trying to get a job, but you are facing discrimination, and sometimes you do not even know you are facing that, and so you are stressed about that… I do not know. I could go on this forever. I’m lost for words right now because I think it’s systemic but also like misinformation, which can also be systemic but also like interpersonal… just not thinking of sleep as like a health-related priority. So, like sleep in the psyche of black people, again having to do with the overworked systemic kind of things, I think is not a priority in health and wellness…”.

Participants were also questioned about the challenges black individuals encounter in relation to sleep. The vast majority highlighted external factors, including chronic stress, financial obligations, housing insecurities and neighborhood environments, work-life balance, household responsibilities, and cultural background as significant barriers to obtaining quality sleep (Figure 10). These external influences, as identified by participants, adversely affect black individuals. For example, participants reported:

*“[We] tend to worry a lot about the cares of life. How are we gonna pay our bills financially? How are we going to take care of taking our child to the doctor if we don’t have any more sick days. How are we going to get things done? How am I going to make sure my child gets a good meal when I’m so tired I can’t stand up?”*

*“There’s a lot about chronic stress having biological effects on your body. I mean for many different reasons people can be stressed out. That has nothing necessarily to do with their race. But I think there tends to be everyday stressors associated with race regardless of what you do in your life career wise or whatever your day to day looks like…. Maybe the neighborhood, the noise, the commotion, and the movement outside… I know here in my neighborhood… that kinda disturbs my sleep at night.”*

## 4 Discussion

Inadequate sleep (IS) is linked to an increased risk of chronic diseases which are a leading cause of death in the US (1, 8, 23). TCIM, incorporating both modern and traditional approaches, has been recognized as a means to enhance sleep health outcomes (17). Despite this recognition, there is a literature gap in understanding the impact of TCIM among the black population, which faces a disproportionate burden of IS and chronic diseases. Previous research indicates that typical health lifestyle behaviors may not have the same benefits for Black individuals compared to White individuals (21, 22). Therefore, adapting TCIM interventions for Black individuals without considering their unique biopsychosocial pathways and experiences may be ineffective. Photovoice, a qualitative methodology rooted in community-based participatory research principles, involves engaging community members in producing culturally informed results through interviews and digital media (32, 33, 38, 44). This study adopts an asset-based model of current TCIM practices, followed by an exploration of facilitators and empowerment using the SHOWeD photovoice protocol and the PEN-3 Cultural Model (33, 35).

Our research, focusing on black participants (N = 25) with a history of IS, revealed insightful results through qualitative content analysis. The sample of heterogenous Black individuals, averaging 37 years of age and representing diverse backgrounds, highlighted a range of culturally influenced perspectives and practices affecting their sleep. Five key themes emerged from the analysis: (1) Natural Wellness, emphasizing the use of natural sleep aids and herbal remedies; (2) Self-Care, focusing on personal maintenance and relaxation techniques; (3) Leisure, where pets and recreational activities played a significant role in relaxation; (4) Mental Stimulation, involving mindfulness and reading as preparatory steps for sleep; and (5) Spiritual Wellness, highlighting the importance of faith-based practices in achieving tranquility. Participants underscored the role of cultural heritage in shaping their sleep health and related behaviors. There is huge heterogeneity within black group of how sleep perceived which also sheds light into varying degrees of sleep health literacy. Notably, Caribbean participants discussed the use of herbalism and plant medicine as integral to their sleep routines, reflecting deep-rooted cultural practices. Self-identified African American or black participants pointed out lifestyle and dietary habits influenced by cultural upbringing as factors impacting sleep health. Participants also shared the cultural expectations in familial roles and responsibilities around productivity and sleep, revealing a spectrum of views within diverse US Black individuals. The research also touched upon the role of mental health therapy, particularly among immigrant participants, highlighting its effectiveness in alleviating stress and improving sleep.

### 4.1 Implications for practice

Applying the PEN-3 Cultural Model provides a multifaceted approach that acknowledges the unique cultural, familial, and societal aspects when addressing insufficient sleep within the US black communities. The diversity in US Black individuals constitutes a plethora of cultural heritages influencing health knowledge, attitudes, and behaviors. The critical need for cultural sensitivity and personalization in interventions is evident, especially given the prevalence of sleep-related issues among this demographic (3, 46, 47). Interventions should be tailored to accommodate the diverse cultural backgrounds within these communities. This includes considering natural remedies, spiritual practices, and leisure activities that resonate with diverse Black individuals’ cultural heritage and nuanced contexts. Furthermore, engagement of families and communities is crucial in addressing sleep health among black communities (48). Sleep health programs must recognize the influence of family dynamics and community norms on sleep behaviors, which are particularly salient in black communities (9, 48). Incorporating these broader social structures, interventions can leverage existing support systems, thereby enhancing the effectiveness and sustainability of behavioral changes.

Education and awareness around the importance of sleep and its related factors, such as diet, exercise, and mental health, are essential components of these interventions (25). Given the disparities in sleep health and access to sleep-related health care in black communities, culturally relevant and accessible educational programs are vital (4, 9, 48). Such initiatives can empower individuals with the knowledge to make informed decisions about their sleep health. Furthermore, addressing mental health is another key aspect, particularly considering the relationship between stress, mental health, and sleep in US Black individuals (26). Access to mental health services, including therapy and counseling for vulnerable populations such as black women and immigrants (49), can play a significant role in improving sleep health. These services can help mitigate the stressors uniquely experienced by Black individuals, thereby improving overall sleep quality.

The study sample exhibited diversity within the large racial category of “black.” Results underscored the heterogeneity within black ethnicities which is often veiled due to aggregate data at the black race level. With the exponential growth in Caribbean, African, and other Black groups in the United States (US) due to immigration and birthrates, the label “black” has come to encompass an increasingly heterogeneous group with important implications for the study of health disparities (50, 51). For example, we know chronic diseases such as CVD and T2DM present differently by race/ethnicity, sex, and age, with older black women at highest risk for poor outcomes (52–55). However, limited studies have focused on the underlying biopsychosocial factors driving this health inequity such as nuanced ethnocultural knowledge, attitudes, and behaviors. Furthermore, cultural and spiritual influences play pivotal roles in health outcomes, particularly within black populations, extending beyond conventional biopsychosocial models (48). These factors deeply influence beliefs, behaviors, and resource access, shaping preferences for healthcare modalities. TCIM approaches hold significance, reflecting cultural heritage and spiritual practices in managing health (12, 56). For instance, supportive networks within cultural and spiritual communities offer crucial emotional aid and a sense of belonging, augmenting overall well-being. Additionally, coping mechanisms like prayer or meditation, rooted in cultural and spiritual beliefs, aid in stress management (57–60). These frameworks foster resilience and imbue life with meaning, enhancing psychological health (61–64). Recognizing and comprehending these dynamics are imperative for delivering culturally sensitive healthcare tailored to the unique needs of black populations, ultimately promoting holistic well-being. Findings from this study identify relevant sociocultural themes from a qualitative standpoint to help elucidate intervention targets. Future studies should consider how exposure to, and appraisal of stress may vary by race/ethnicity (e.g., reason for immigration, context of reception, and cultural perspectives), and in turn, pathways of psychogenic stress may also vary accordingly for tailored intervention (62, 65, 66).

### 4.2 Conclusion

Public health policies must consider the unique needs of diverse black communities when designing sleep health programs. This entails not only recognizing the specific challenges faced by these communities but also dedicating resources to community-specific research and intervention programs (67). Such policy initiatives can bridge the gap between research findings and practical, community-based solutions, leading to more effective and inclusive sleep health programs. Addressing IS in black communities through the lens of the PEN-3 Model requires a comprehensive strategy that integrates cultural sensitivity, family and community dynamics, education, mental health support, and informed policymaking. This approach, grounded in a deep understanding of the specific challenges and needs black communities, offers a pathway to more effective and sustainable interventions in sleep health. For instance, addressing equity concerns related to issues such as housing insecurities, job wages, and neighborhoods is crucial and requires adequate attention from stakeholders in positions that influence systemic structures (4, 23, 48).

### 4.3 Strengths and limitations

Individuals began by sharing their initial experiences of obstacles in a private setting to reduce response bias and encourage active participation. The research methodology involved subsequent focus group interviews to validate findings and elaborate on perceptions of TCIM. Conducted in Miami, a city renowned for its cultural diversity and representation of various ethnic groups of Black individuals, the study aimed to offer insights applicable to diverse contexts. Importantly, the study did not encompass advanced knowledge assessment, behavior change evaluation, or clinical intervention. Despite these limitations, the study’s outcomes contribute valuable information for optimizing public health planning and delivery strategies.

## Data availability statement

The raw data supporting the conclusions of this article will be made available by the authors, without undue reservation.

## Ethics statement

The studies involving humans were approved by University of Miami Institutional Review Board. The studies were conducted in accordance with the local legislation and institutional requirements. The participants provided their written informed consent to participate in this study. Written informed consent was obtained from the individual(s) for the publication of any potentially identifiable images or data included in this article.

## Author contributions

RM: Conceptualization, Data curation, Formal analysis, Funding acquisition, Investigation, Methodology, Project administration, Resources, Software, Supervision, Validation, Visualization, Writing – original draft, Writing – review & editing. MC: Data curation, Formal analysis, Investigation, Methodology, Software, Validation, Writing – original draft, Writing – review & editing. MW: Data curation, Formal analysis, Investigation, Methodology, Software, Validation, Writing – original draft, Writing – review & editing. FZ: Funding acquisition, Project administration, Resources, Writing – review & editing. AS: Funding acquisition, Project administration, Supervision, Writing – review & editing. GJ-L: Data curation, Funding acquisition, Investigation, Resources, Supervision, Writing – review & editing.
